# Hematological Parameters in Individuals with Beta Thalassemia Trait in South Sumatra, Indonesia

**DOI:** 10.1155/2022/3572986

**Published:** 2022-05-05

**Authors:** Dian Puspita Sari, Pustika Amalia Wahidiyat, Iswari Setianingsih, Ina S. Timan, Djajadiman Gatot, Aria Kekalih

**Affiliations:** ^1^Department of Child Health, Mohammad Hoesin Palembang Hospital, Palembang, Indonesia; ^2^Department of Child Health, Faculty of Medicine University Indonesia, Dr. Cipto Mangunkusumo Hospital, Jakarta, Indonesia; ^3^Eijkman Institute for Molecular Biology, Jakarta, Indonesia

## Abstract

**Background:**

*β*-Thalassemia has a very wide clinical variation, depending on the severity of the patient's condition. Individuals with *β*-thalassemia traits are usually asymptomatic; however, laboratory examination will show mild anemia with microcytic hypochromic erythrocytes morphology with wide variation depending on the genotype. This study was conducted to determine the reference value of hematological parameters and hemoglobin (Hb) analysis based on the phenotype of *β*-thalassemia (*β*^0^ and *β*^+^) and determine the differences of hematological characteristics between the two phenotypes.

**Methods:**

This cross-sectional study was conducted by evaluating the hematological parameters and Hb analysis of the *β*-thalassemia trait in the family of thalassemia patient population. The subjects were divided into *β*^0^ and *β*^+^. The subject with normal Hb analysis with or without iron deficiency was excluded.

**Results:**

A total of 203 subjects with thalassemia traits were included from the families of thalassemia patients, consisting of 101 subjects with *β*^0^-thalassemia, 82 subjects with *β*^+^-thalassemia, and the mutation had not been found in 20 subjects. There was a relationship in the mean/median of hematological parameters, HbA_2_ and HbF, between *β*^0^-thalassemia and *β*^+^-thalassemia (*P* < 0.05). ROC for each hematological parameter, HbA_2_ and HbF, showed that the highest diagnostic value based on the area under the curve was mean corpuscular hemoglobin (MCH) (0.900) and mean corpuscular volume (MCV) (0.898). The cutoff point of MCH for *β*^0^-thalassemia trait was ≤20.5 pg (sensitivity 85%, specificity 90%) and MCV was ≤66.8 fL (sensitivity 87%, specificity 87%).

**Conclusion:**

MCH values can be used as a screening tool for predicting *β*^0^-thalassemia in the relatives of thalassemia patients in the South Sumatra population.

## 1. Introduction

Thalassemia is an autosomal recessive genetic disorder characterized by hemolytic anemia. *β*-Thalassemia is caused by abnormalities of the single *β*-globin gene that interfere with the synthesis of the globin chains of hemoglobin; this interference can cause total (*β*^0^) or partial (*β*^+^) disruption of globin chain synthesis [1, 2]. Currently, thalassemia is the most widespread monogenic disease in the world. Based on the WHO, in 2008, at least 5.2% of the world's population were thalassemia carriers, 1.1% of which were couples that had a risk of having children with hemoglobin disorders [3]. The prevalence of trait carriers was found to be very high in the populations of Africa, Southeast Asia, the Eastern Mediterranean, and the Western Pacific [4]. Southeast Asia is the region with the most complex thalassemia genotype compared to other countries. South Sumatra is one of the areas with the highest carrier rates for hemoglobinopathies in Indonesia, with the prevalence being 15%, consisting of 9% carriers of *β*-thalassemia trait and 6% HbE [5].

*β*-Thalassemia has a very wide clinical variation, depending on the severity of the patient's condition and age at the time of diagnosis. Thalassemia traits is asymptomatic; however, laboratory examination will show variation of Hb levels; it could be normal to up to 2 g/dL, with microcytic hypochromic erythrocytes [6]. Hemoglobin analysis will demonstrate an increase in HbF and HbA2 levels. The Indonesian thalassemia management guidelines recommend the routine use of erythrocyte index as a screening tool in patients with thalassemia. Based on this guideline, patients with MCV <80 fL and MCH <27 pg should be evaluated further. In Indonesian Guideline for Management of Thalassemia (2018), possibility of thalassemia traits is identified by HbA_2_ examination: an HbA_2_ level between 3.6 and 4.2% suggests mild *β*^+^-thalassemia and levels between 4 and 9% suggest heterozygote *β*^0^-thalassemia and severe *β*^+^-thalassemia [7]. This study was conducted to determine the appropriate reference value of hematological parameters and Hb analysis based on the phenotype of *β*-thalassemia (*β*^0^ and *β*^+^) and determine the differences of hematological characteristics between the two phenotypes.

## 2. Methods

The cross-sectional study was conducted at the Thalassemia Center in Mohammad Hosein General Hospital (RSMH), Palembang, from October 2020 to December 2020. RSMH is the tertiary hospital which cover the South Sumatera region. The ethical clearance of this study was approved by RSMH (Letter No.: 111/kepkrsmh/2020) and RSCM-FKUI (Letter No.: KET-1369/UN2.F1/ETIK/PPM.00.02/2020) Ethical Committee. Research subjects are parents and siblings of thalassemia patients who routinely receive blood transfusions. The method uses consecutive sampling; subjects were recruited until the minimum sample size was reached, until it meets the number of samples, or until the end of the study.

Written consent was obtained from all subjects who were willing to participate. Subjects were enrolled consecutively from relatives of patients who received regular blood transfusion in RSMH. For children under 18 years of age, consent was obtained from their parents. The screening was carried out in the form of interviews, physical examination, and blood collection for hematological examination, Hb analysis, and DNA analysis. Interviews were conducted to determine the identity, ethnic origin, and history of transfusion. The ethnic groups were grouped based on the origin of the South Sumatran and non-South Sumatran tribes. Physical examination included an examination for clinical symptoms such as pallor and splenomegaly. 15 mL of blood was collected intravenously for hematological examination, Hb analysis, iron status, and DNA analysis. We excluded the subjects with normal Hb analysis with or without iron deficiency. Hb analysis was normal if HbA_2_ is <3.5%, HbF <1%, and HbA >97% [7]. Iron deficiency was defined if ferritin serum was <12 ng/mL for under 5 years old subjects and <15 ng/mL in subjects ≥5 years old [8].

DNA analysis was not performed on subjects who had normal Hb analysis or evidence of iron deficiency. *β*-Thalassemia mutation type was determined by DNA analysis using the PCR-RFLP technique followed by DNA sequencing. Examination of mutations in exons 1 and 2 and intron 1 that often appears in Indonesia was carried out on all subjects. In subjects whose mutations were unidentified, the examination was continued on exon 3. The types of globin gene mutation were grouped according to the *β*-globin synthesis phenotype. *β*^0^-Thalassemia is the phenotype of a mutation that causes the *β*-globin chains do not produce, such as mutations in IVS1-nt5 (G ⟶ C), IVS1-nt1 (G ⟶ T), CD15 (TGG ⟶ TAG), CD17 (AAG ⟶ TAG), CD30 (+C), CD 35 (−C), CD41-42 (−TCTT), CD8/9 (+G), IVS1-nt2 (T ⟶ C), and CD 26 (GAG ⟶ TAG), whereas in *β*^+^-thalassemia, *β*-globin chains were formed, but has declined in function, which included the mutations in CD 19 (AAC ⟶ AGC) and CD-26 (GAG ⟶ AAG)/HbE. [9].

### 2.1. Statistical Analysis

Descriptive data in the form of mean, median, and range were used to describe the distribution of numerical data (Hb, erythrocyte index, Hb analysis). Data analysis was conducted to determine the relationship between thalassemia mutation phenotype with Hb levels, MCV, MCH, MCHC, RDW, and Hb analysis using an independent *t*-test. The assessment of the cutoff point of each variable that had a relationship was using ROC curve. The diagnostic value (sensitivity, specificity, positive predictive value, negative predictive value, likelihood ratio positive, and likelihood ratio negative) was calculated. The analysis was carried out using the SPSS version 20 for Windows.

## 3. Results

There were 224 subjects in the current study, consisting of 203 subjects who were carriers based on Hb analysis and DNA analysis and 21 subjects who had normal Hb analysis, in which 3 had iron deficiency anemia.

The carriers consisted of 154 subjects who are parents, 24 subjects are siblings, 4 subjects are uncles, and 1 subject is a cousin of thalassemia patients who has routinely received transfusions at RSMH. The characteristics of the subjects are given in Table 1. Most of the subjects carrying the trait were females (64.5%); the median age was 37.3 (2.3–64.5) years and came from the South Sumatran ethnic group (70.4%). Most of the subjects lived outside of Palembang city (56.7%). Based on DNA analysis, 20 subjects had no mutation identified yet, but the hematologic features, Hb analysis, and iron status suggested to thalassemia condition, consisting of 18 subjects are parents and 2 subjects are siblings.

The type of mutation grouped by the *β*-globin chain synthesis is given in Table 2. We identified two types of heterozygotes, *β*^+^-thalassemia mutations and 10 types of *β*^0^-thalassemia mutations. The most common mutations found were mutations in IVS1-nt5 heterozygotes in 59 (32.2%) subjects, followed by HbMalay heterozygotes in 47 (25.7%) subjects, HbE heterozygotes in 35 (19.1%) subjects. IVS1-nt1 heterozygotes in 16 (8.7%) subjects, CD 41–42 (−TCTT) heterozygotes in 14 (7.7%) subjects, CD 8/9(+G) heterozygotes in 4 (2.2%) subjects, CD 35(−C) heterozygotes in 3 (1,6%) subjects, and CD15, CD26 (G ⟶ T0), and CD 30 heterozygotes mutation each in one subject (2.5%).

Table 3 provides hematological parameters and Hb analysis by type of mutation. There were statistically significant differences in the mean/median Hb, MCV, MCH, MCHC, RDW, HbA_2_, and HbF, where Hb, MCV, MCH, and MCHC levels were lower in *β*^0^-thalassemia compared to *β*^+^-thalassemia. Meanwhile, the RDW level was higher. Besides, based on Hb analysis, HbA_2_ and HbF levels were higher in *β*^0^-thalassemia compared to *β*^+^-thalassemia with a *P* value <0.05.

Table 4 provides the hematological parameters and Hb analysis of each genotype (CD 35 (−C), CD 41–42 (−TCTT), CD8/9 (+G), IVS1-nt1, IVS1-nt5, HbE, and HbMalay). HbE and HbMalay was frequent mutation in South East Asia region; this study showed that Hb, MCV, and MCH level of HbE trait was greater than HbMalay trait, while the RDW was lower.

The diagnostic values (sensitivity, specificity, positive and negative predictive value, likelihood ratio positive and negative, and characteristic ROC) in connection with the cutoff in this population for differential diagnosis of *β*^0^-thalassemia compared to *β*^+^-thalassemia are given in Table 5.

**Figure 1:**
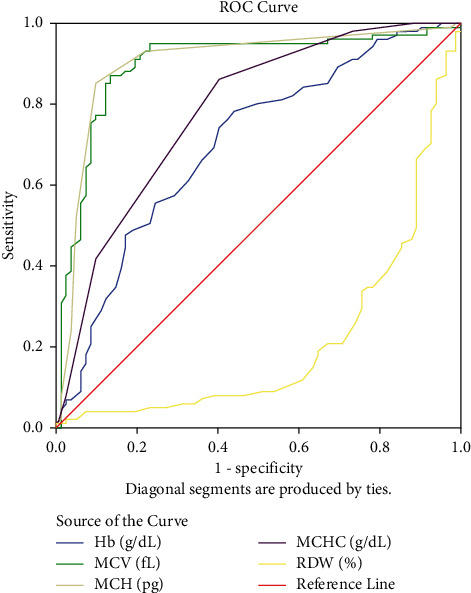
Receiver operative characteristic curves (ROC) of hematological parameters (Hb, MCV, MCH, MCHC, and RDW) (*P* value of each formula <0.001).

**Figure 2:**
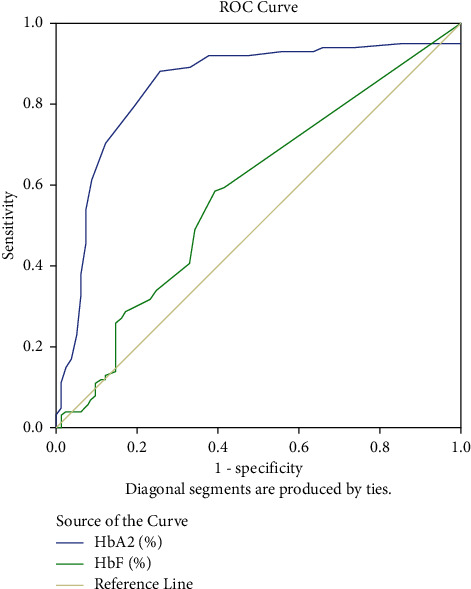
Receiver operative characteristic curves (ROC) of HbA_2_ (*P* < 0.001) and HbF (*P*=0.053).

Figure 1 shows ROC for hematological parameter calculation, and Figure 2 shows ROC for Hb analysis calculation. From Table 5, Figures 1 and 2, the highest diagnostic value was related to MCH to differentiate *β*^0^-thalassemia compared to *β*^+^-thalassemia. There was no significant difference in the AUC of HbF levels between these types of mutation.

## 4. Discussion

Southeast Asia region has the most complex genotypes of thalassemia in the world as the result of various combinations of globin chain gene mutations from interethnic marriage [10]. South Sumatra has a high carrier prevalence of *β*-thalassemia trait as much as 15% [11], so that screening is necessary to prevent the birth of children with thalassemia major. In the current study, analysis of 183 subjects was conducted to compare the hematological parameters between *β*^0^-thalassemia compared to *β*^+^-thalassemia carriers in the families of thalassemia patients to determine the predictive value that can be used as a screening tool. The current study found 12 types of mutations in 183 subjects, with the most common mutations being IVS1-nt5 (32.2%), followed by HbMalay (25.7%) and HbE (19.1%). A previous study in 2003 that examined trait-carrying mutations in the Malay ethnicity of South Sumatra in the general population found that the most common mutations were HbE (36.3%), followed by HbMalay (34.09%) and IVS1-nt5 (9.09%) [5].

Erythrocyte index (MCV and MCH) is a parameter used in screening for thalassemia, based on the International Thalassemia Federation (TIF) guidelines. Levels of MCV <78 fL and MCH <27 pg, with peripheral blood features of microcytic, hypochromic, and anisopoikilocytosis can be suspected as carriers [1]. Based on Indonesia Guidelines for Management of Thalassemia, the suspected carriers in general population, if the MCV value was <80 fL and the MCH value was <27 pg [7].

In the current study, the mean of Hb, MCV, MCH, and MCHC in *β*^0^-thalassemia was lower than *β*^+^, besides the RDW, HbA_2,_ and HbF levels were higher. These data could explain the differences between the two phenotypes to define cutoff values for each variable. Indonesia, especially South Sumatra, have various ethnic groups. The cutoff points of hematological parameters and Hb analysis can be useful to differentiate the type of thalassemia mutation in the carrier population in South Sumatra for screening purposes in the families of thalassemia patients. Evaluation of hematological parameters and Hb analysis in this study was performed and compared according to the ROC curve. The result shows that MCH has the largest AUC (0.900).

Hematological characteristics of *β*-thalassemia heterozygotes have wide variation based on the type of mutation [12, 13]. Baliyan et al.' [14] study in India showed that MCV <74 fL combined with MCH <28 pg can be used as a cutoff for the screening test of thalassemia in antenatal anemic woman in South Asian region, if the HPLC in the facility does not exist (sensitivity 95%, specificity 16%). Another study in Iran showed that MCH <27 pg was more sensitive compared with MCV <80 fL for screening of *β*-thalassemia traits in the general population [15]. The study in Israel [16] showed that there was a relationship between MCH and type of mutation with cutoff point 20.94; however, MCH was less sensitive to differentiate between *β*^0^ and *β*^+^ populations. This present study found that MCH value ≤ 20.5 pg was predicted as *β*^0^-thalassemia (sensitivity 85%, specificity 90%, PPV 91%, NPV 83%, and likelihood ratio positive 8.73).

A study in Israel showed that MCV lower than 66.96 fL could predict the possibility of *β*^0^ mutation (sensitivity 77% and specificity 91%) [16]. Almost similar to this present study, we found the cutoff point of MCV was ≤66.8 fL (sensitivity 87%, specificity 87%) for *β*^0^ and >66.8 for *β*^+^.

The RDW examination is usually done to differentiate between iron deficiency anemia and thalassemia independent of transfusion. The cutoff > 14% suggests thalassemia trait carriers [17]. In the present study, we found significant differences in the levels of RDW between *β*^0^-thalassemia and *β*^+^-thalassemia with *P* < 0.05. The median RDW is known to be lower in *β*^0^-thalassemia compared to *β*^+^-thalassemia. In the study done in Medan, North Sumatra (2019) [18], the RDW level in carriers of *β*-thalassemia trait was between 15.7% and 16.5%, while this present study has a wider range of RDW which was 12–20.8%.

Based on Hb analysis, we found that there are significant differences between the levels of HbA_2_ and HbF in *β*^0^-thalassemia and *β*^+^-thalassemia, where the levels of HbA_2_ in *β*^0^-thalassemia were between 1.7 and 6.6% with a median of 5.1%. The lower levels found in the current study compared to the threshold used by the guidelines used for the screening of thalassemia in which levels of HbA_2_ in thalassemia suggestive of *β*^0^-heterozygotes were 4–9% and in *β*^+^-thalassemia between 2.9 and 6% with a median of 4.3%. HbA_2_ levels can also be affected by iron status, whereas in deficiency anemia, HbA_2_ levels will decrease [7]. The HbA_2_ level used in screening to detect thalassemia is >3.5%. However, in this study, 6.6% of subjects had HbA_2_ < 3.5%. We calculated the cutoff point of HbA_2_ of *β*^0^-thalassemia in this study using the ROC curve, finding that HbA_2_ level ≥ to 4.65% was predicted to be *β*^0^-thalassemia, with sensitivity 88% and 74% of specificity. The HbA_2_ value in this subject could be affected by iron status; there were 5 subjects who had iron deficiency, and those are 2/101 subjects with *β*^0^-thalassemia and 3/82 subjects with *β*^+^-thalassemia.

From the current study, we concluded that MCH can be used as a screening tool to differentiate the type of *β*-thalassemia mutation (*β*^0^ or *β*^+^) in populations of carriers consisting of relatives of patients with thalassemia in South Sumatra.

## Figures and Tables

**Table 1:** Characteristics of the subject of the trait carrier (*n* = 203).

Characteristics	*N*	%

Age group (years)		
2–10	15	7.4
>10	188	92.6

Gender		
Male	72	35.5
Female	131	64.5

South Sumatra ethnic group		
Yes	143	70.4
Not	60	29.6

Mutation type		
*β*^0^-Thalassemia heterozygotes	101	49.7
*β*^+^-Thalassemia heterozygotes	82	40.3
The mutation was not found^†^	20	10

^†^Mutation was not found in exon 1, exon 2, exon 3, and several introns that are often populated by Indonesia.

**Table 2:** Types of *β*-thalassemia mutations found in the present study (*n* = 183).

Mutation type	*N* = 183	%

*β* ^0^-Thalassemia heterozygote		
IVS1-nt5 (G ⟶ C)	59	32.2
IVS1-nt1	16	8.7
CD 41–42 (−TCTT)	14	7.7
CD 8/9 (+G)	4	2.2
CD 35 (−C)	3	1.6
CD 15	1	0.5
CD 26 (G > T)	1	0.5
CD 30	1	0.5
CD 71/72 (+A)	1	0.5
IVS1-nt2	1	0.5
*β* ^+^-Thalassemia heterozygote		
HbMalay	47	25.7
HbE	35	19.1

**Table 3:** Hematological variables among *β*^0^ and *β*^+^-thalassemia carrier.

Variable	*β* ^0^-Thalassemia (*N* = 101)	*β* ^+^-Thalassemia (*N* = 82)	*P* value

Hematological parameter			
Hb (g/dL)^*∗*^	11.4 ± 1.2	12.4 ± 1.4	<0.001^‡^
MCV (fL)^*∗∗*^	63.6 ± 4.8	72.05 ± 5.3	<0.001^‡^
MCH (pg)^*∗∗*^	19.5 ± 1.8	23.1 ± 2.3	<0.001^‡^
MCHC (g/dL)	30.7 ± 0.9	31.9 ± 1.4	<0.001^‡^
RDW (%)^*∗∗*^	17.4 ± 1.7	15.6 ± 1.7	<0.001^‡^

Hemoglobin analysis			
HbA_2_ (%)^*∗∗*^	5.02 ± 0.8	4.3 ± 0.6	<0.001^‡‡^
HbF (%)^*∗∗*^	0,91 ± 1,8	0.8 ± 2.8	0,040^‡‡^
HbA (%)^*∗∗*^	93.7 ± 3.05	85.9 ± 11.6	0.078^‡‡^

^*∗*^Normal distribution,^*∗∗*^abnormal distribution, ^‡^Independent *t*-test,^‡‡^Mann–Whitney test.

**Table 4:** Distribution of hematological parameters and Hb analysis for each genotype.

Genotype	*β* ^0^ heterozygotes				*β* ^+^ heterozygotes		
	CD 35 (−C)	CD 41–42 (−TCTT)	CD 8/9 (+G)	IVS1-nt1	IVS1-nt5	HbE	HbMalay
	*n* = 3	*n* = 14	*n* = 4	*n* = 16	*n* = 59	*n* = 35	*n* = 47

Hematological parameter							
Hb (g/dL)^*∗∗*^	12.1 (10.8–12.3)	10.9 (8.8–13.5)	12.1 (11.1–13.0)	11.0 (9.4–13.3)	11.4 (9.3–14.6)	12.7 (9.5–15.1)	12.3 (8.3–15.5)
MCV (fL)^*∗∗*^	65.9 (63.3–68.3)	60.2 (55.6–63.4)	61.3 (61.1–61.5)	62.5 (54.4–85.3)	63.9 (51.9–79.5)	75.2 (61.9–83.3)	71.2 (53,4–81,3)
MCH (pg)^*∗∗*^	20.0 (20.0–21.0)	18.5 (17.0–20.0)	18.0^a^	19.0 (17.0–28.0)	20.0 (15.0–26.0)	24.0 (18.0–30.0)	22.0 (16.0–25.0)
RDW (%)^*∗∗*^	15.6 (14.5–17.7)	18.0 (15.4–20.8)	18.2 (17.6–19.1)	17.5 (12.1–20.1)	17.2 (12.6–20.2)	14.6 (12.3–18.3)	15.6 (13.5–20.6)

Hemoglobin analysis							
HbA2 (%)^*∗∗*^	5.4 (5.1–5.4)	5.5 (4.9–6.6)	4.8 (4.7–5.3)	5.2 (2.7–6.1)	5.0 (1.9–6.4)	3.7 (2.9–5.7)	4.5 (3.7–6.0)
HbF (%)^*∗∗*^	0 (0–1.4)	0.4 (0–2.9)	0.7 (0–0.4)	0.7 (0–4.1)	0 (0–12.7)	0 (0–24.5)	0.2 (0–3.9)
HbA (%)^*∗∗*^	94.6 (93.2–94.9)	93.9 (90.5–95.1)	95.2 (94.3–95.3)	93.6 (90.7–96.8)	94.6 (70.9–98.1)	71.1 (68.0–94.8)	95.1 (91.4–96.3)
HbE (%) (*n* = 35)^*∗∗*^					1 subject: 25.4	25.2 (23.0–27.0)	

^*∗∗*^Abnormal distribution (median). ^a^MCH value in CD 8/9(+G) mutation has the constant value (4 subjects) = 18 pg.

**Table 5:** Evaluation of different hematological parameters and Hb analysis in the differentiation of *β*^0^-thalassemia compared to *β*+-thalassemia.

	Sensitivity	Specificity	PPV (%)	NPV (%)	LR +	LR −	Cutoff for *β*^0^ in our population	AUC (95% CI)

Hematology parameter								
Hb	78	56	69	68	1.78	0.39	≤12.3	0.702 (0.625–0.778)
MCV	87	87	89	85	2.71	0.29	≤66.8	0.898 (0.847–0.948)
MCH	85	90	91	83	8.73	0.16	≤20.5	0.900 (0.849–0.952)
MCHC	86	60	73	78	2.14	0.23	≤31.5	0.784 (0.716–0.851)
RDW	79	71	77	73	0.29	2.71	≥16.15	0.211 (0.142–0.280)

Hemoglobin analysis								
HbA_2_	88	74.4	81	84	3.44	0.16	≥4.65	0.844 (0.781–0.906)
HbF	58	61	65	54	1.50	0.68	≥0.35	0.583 (0.500–0.667)

PPV, positive predictive value; NPV, negative predictive value; LR, likelihood ratio; AUC, area under the curve.

## Data Availability

The data used to support this study are available from the corresponding author upon request.
